# Retrieval Practice, with or without Mind Mapping, Boosts Fact Learning in Primary School Children

**DOI:** 10.1371/journal.pone.0078976

**Published:** 2013-11-12

**Authors:** Stuart J. Ritchie, Sergio Della Sala, Robert D. McIntosh

**Affiliations:** Human Cognitive Neuroscience, Department of Psychology, University of Edinburgh, Edinburgh, United Kingdom; The Ohio State University, Center for Cognitive and Brain Sciences, Center for Cognitive and Behavioral Brain Imaging, United States of America

## Abstract

Retrieval practice is a method of study in which testing is incorporated into the learning process. This method is known to facilitate recall for facts in adults and in secondary-school-age children, but existing studies in younger children are somewhat limited in their practical applicability. In two studies of primary school-age children of 8–12 years, we tested retrieval practice along with another study technique, mind mapping, which is more widely-used, but less well-evidenced. Children studied novel geographical facts, with or without retrieval practice and with or without mind mapping, in a crossed-factorial between-subjects design. In Experiment 1, children in the retrieval practice condition recalled significantly more facts four days later. In Experiment 2, this benefit was replicated at one and five weeks in a different, larger sample of schoolchildren. No consistent effects of mind mapping were observed. These results underline the effectiveness of retrieval practice for fact learning in young children.

## Introduction

Students tend to believe that the best way to learn new facts is by prolonged or repeated exposure [1], but a body of evidence in cognitive psychology attests to the value of retrieval practice for boosting learning [2], [3], [4], [5]. This is sometimes known as the ‘testing effect’, because the critical feature is that studying should include test periods, during which the student tries to recall the facts without checking the source material.

Whereas most experimental evidence for retrieval practice comes from adult participants tested in laboratory settings, a number of studies have applied the technique in school classrooms, mostly with children aged 11 years and above [6], [7], [8], [9], [10]. These studies have shown that retrieval practice facilitates learning of a variety of materials in the classroom. For instance, Roediger et al. [9] showed that retrieval practice can be incorporated into the school curriculum, with low stakes quizzing at various points throughout the term showing learning benefits on later exams in US sixth grade children (aged 11–12).

Some experiments have also demonstrated benefits of retrieval practice in samples including younger children. An early experiment [11] had children aged 6–14 years learn nonsense syllables and biographical material, with varying amounts of time devoted to self-testing by silent recitation. As self-testing time increased, so did the amount of material recalled three to four hours later. Much more recently, in two experiments [12], both with twenty-eight children aged 9–11, groups who were tested immediately after learning fictional map locations had better recall for the locations one day later than a ‘study only’ group, and the testing effect transferred to questions more complex than those on the immediate test (for other experiments including young children, see [13], [14], [15]).

However, these studies have a number of limitations to their practical applicability. They have tended to focus on small samples [12], [14], [15], to use short testing intervals [11], [12], or to test relatively simple materials such as word lists [13]. To our knowledge, no one study has addressed these limitations in a sample that includes children of primary school age (normally up to 11/12 years of age in the UK). The present study tests the effect of retrieval practice in two reasonably large samples of children, across intervals of up to five weeks, on educationally-relevant geographical facts.

Despite its growing evidence base, retrieval practice is used rarely in schools [4], especially by comparison with some other less well-evidenced techniques. For instance, mapping techniques have become popular in classrooms worldwide [16]. These include ‘mind-mapping’, the drawing of diagrams to organize facts into categories, and the more sophisticated ‘concept-mapping’, in which the diagram visually represents the inter-relations between facts. Proponents claim that individuals with a ‘visual’ learning style benefit from these techniques [17]. However, a recent review concluded that there is no good evidence for the claim that a student’s preferred ‘learning style’ influences their learning outcome from different instructional techniques [18].

In addition, the evidence regarding the effect of mind mapping on learning is sparse and mixed. One study [19] reported a benefit of mind mapping on fact learning in medical students, but other studies in similar groups have found no effect [20], [21]. An encouraging finding in a younger population (sixty-two 13–14 year olds) was that the use of mind maps throughout a science course yielded higher scores on a later test than did standard note-taking [17]. We are aware of no similar studies in children of primary school age, despite the fact that mind mapping is very commonly used with younger students.

One prior study [22] has incorporated both mapping *and* retrieval practice. Undergraduate students who were given an immediate test on facts they had studied had better recall at one week than did those who studied the facts once, studied them repeatedly, or who drew a concept map. The authors concluded that retrieval practice was superior to concept mapping (for further discussion of this result, see [23], [24]). Even so, it should be emphasized that retrieval practice does not preclude mapping techniques, and it is possible that their combination (e.g. self-testing using a mapping technique) would be more beneficial than either technique alone. This potential to combine techniques was noted by the authors [22], while Roediger [25] has discussed the need for studies on combinations of learning techniques.

This study, like that of Karpicke and Blunt [22], tested the effects of retrieval practice and a mapping technique on fact learning, but with three major differences. First, we focused on much younger participants, primary school children aged 8–12 years. Second, for this age group we used simple mind mapping (with which the children were already familiar) rather than more complex concept mapping. Third, we used a crossed-factorial design to test not only the effects of retrieval practice and mind mapping, but also their combination. In Experiment 1, we tested the effects of these techniques in a sample of 109 children, within a school week. In Experiment 2, we replicated our findings in a larger sample, with a longer interval between the learning and test phases, and a somewhat more challenging task.

We hypothesized, consistent with previous work, that retrieval practice should improve memory for facts across time. Given the lack of solid previous evidence, we made no directional prediction regarding mind mapping, or its combination with retrieval practice, which may variously prove to have additive positive effects on memory beyond retrieval practice by allowing the use of a visual encoding strategy, distract from the task at hand and prove detrimental, or make no appreciable learning difference.

## Experiment 1

### Method

#### Ethics statement

Both Experiment 1 and Experiment 2 in the present study were approved by the Psychology Research Ethics Committee at the University of Edinburgh, and written informed consent was obtained from the parent or guardian of each participating child before the experiments began.

#### Participants

Participants in Experiment 1 were 109 pupils (59 female) from Primary 5 and 7 classes (two classes from each year) at Towerbank Primary School, Edinburgh, aged 8–12 years (*M* = 10.29 years, *SD* = 1.07; numbers and ages per class are shown in Table S1).

#### Materials

**Figure 1:**
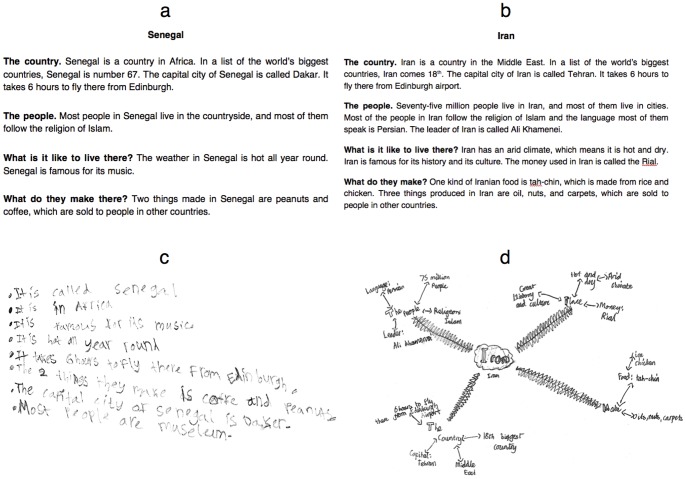
Example factsheets for (a) Primary 4 and (b) Primary 7 in Experiment 2; (c) example notes from one Primary 4 child in Experiment 2 and (d) an example mind map from one Primary 7 child in Experiment 2.

Four single-sided, four-paragraph ‘factsheets’ each concerned a different country likely to be unfamiliar to young children in the UK (Senegal, South Korea, Peru, and Iran). Two example factsheets (from Experiment 2, which had very similar materials; see below) are shown in Figs. 1a and 1b, and the text of all factsheets is provided in the Text S1. Sheets for Primary 5 children had eleven facts (∼one hundred words), and sheets for Primary 7 children had sixteen facts (∼one hundred and thirty words). All factsheets were placed in unmarked envelopes; those to be given to the non-retrieval group also contained a separate blank sheet of paper for note-taking.

#### Learning phase

In the learning phase, children were randomly assigned to a retrieval practice condition within classes (half of the children in each class used retrieval practice), while mind mapping conditions were arranged between classes (one of the two classes from each year used mind mapping). The following paragraphs describe the details of this procedure.

On the Monday of the experimental week, the experimenter visited each classroom and teachers split the classes into two groups (‘retrieval practice’ and ‘non-retrieval’), by running through the class register and assigning successive children to alternating groups (the assortment of children to groups was thus random). The two groups were then seated on opposite sides of the classroom, in sub-groups of four. Within each subgroup, each child was given a different one of the four factsheets. In a few classrooms, where seating required that some subgroups were larger than four, factsheets were handed out so that no child sat directly beside a classmate with the same country. In these larger groups, and in some groups with fewer than four children, factsheets were given out in the order Senegal-South Korea-Peru-Iran to attempt an approximately equal distribution of the four sheets across classrooms and experimental conditions.

The experimenter explained that the children were to learn some facts for a quiz at the end of the week. They were asked to open their envelopes and to read their fact sheet without writing anything. This initial reading period lasted five minutes. For the next five minutes, the children made notes on the facts. Children in one group (non-retrieval) kept the factsheet in view throughout this period and made notes on the blank sheet of paper. Children in the other group (retrieval practice) were required to turn the factsheet over, and to make notes on the blank side. The experimenter and the teacher monitored compliance with instructions.

In half of the classes (one class from each year, randomly selected), the children made notes in the form of a mind map, writing the name of the country in the center of the page, and drawing lines outwards to facts grouped by their categories. All classes at the school regularly used this form of mind mapping to represent facts, such as historical knowledge, or the attributes of characters in reading books. In the other half of the classes, the children were asked to make notes in any way they liked *except* for mind mapping. Examples of mind maps and notes made in this study are shown in Figures 1c and 1d, respectively.

Next, all children read the sheets again for three minutes, without writing anything. Finally, all children continued making notes, as before, for five minutes. The experimenter then collected the sheets. Note that the learning phase thus lasted for 18 minutes overall, but the retrieval practice group had the factsheets visible for only eight minutes, whilst the non-retrieval group had the factsheets visible throughout.

#### Testing phase

On the Friday of the experimental week, the children were given a written recall test, with one question for each fact on their factsheet (all questions, for both year groups, are shown in the Text S1). This test was administered by the experimenter, who read the questions out loud to the whole class. The first question asked the name of the country; the remaining questions were in a fixed, pseudo-random order. Each question was read once, and the children were given as much time as needed to write each answer before the next question. Children were informed they would still gain a mark for misspelled correct responses. The experimenter later scored the tests; since quiz sheets were identical in all four experimental conditions, this scoring was blind to the conditions in which each child participated. Half-marks were awarded for partial, but correct, answers (e.g. “Korea” for “South Korea”; “hot” for “hot and dry”).

### Results

Approximately equal numbers of children learned about each of the four countries (Senegal: thirty-one; South Korea: twenty-eight; Peru: twenty-five; Iran: twenty-five; see Table S2 for the distributions of these sheets across the conditions of the experiment and across the year groups). One sheet from the learning phase was lost, leaving one hundred and eight sheets. For descriptive purposes, scores are given below (and in the tables and figures) in percentage terms; however, *z*-scores were calculated to make the Primary 5 and Primary 7 tests comparable, and were used in all analyses below (re-running the analyses on the percentage data produced near-identical results). Overall, 82.85% of facts were recorded on the note sheets in the learning phase, and a 2×2 ANOVA (retrieval practice condition×mind maps condition) confirmed that there were no significant differences in percentage of facts recorded between conditions [mean difference = 4.95% in favour of no mind maps (*F*(1, 105) = 1.58, *p* = .21); mean difference = .47% in favour of non-retrieval (*F*(1, 105) = .01, *p* = .91)]. Table S3 shows means and standard deviations for performance in the learning phase.

The mean percentage scores on the recall test are shown in Table 1 for each condition; numbers of children per cell of the experiment are shown in Table S4. To test the effects of retrieval practice and mind mapping on fact recall, we ran a 2×2 ANCOVA, with the between-subjects factors of retrieval practice group (retrieval practice vs. non-retrieval) and mind mapping group (mind maps vs. no mind maps). A multiple regression including all potential covariates–age, sex, test/year (Primary 5 vs. Primary 7), country on the factsheets (of the four available), number of facts recorded during the learning phase–indicated that only the number of facts recorded in the learning phase was significantly related to the final test score (*p*<.001; *p*-values for other variables = .50–.94), and thus only this variable was included as a covariate in the ANCOVA. An additional analysis that, instead of using this covariate, scaled the test scores by the number of facts recorded during the initial session, did not appreciably alter the main results reported here.

**Table 1:** Mean percentage scores (SDs) and sample sizes for Experiment 1.

	Mind maps	No mind maps	Total score	*N*
Retrieval	72.09 (21.58)	77.31 (24.22)	74.70 (22.86)	52
Non-retrieval	68.31 (29.80)	64.43 (28.26)	66.23 (28.79)	56
Total score	70.20 (25.83)	70.41 (27.02)	70.31 (26.33)	108
*N*	52	56	108	

Children in the retrieval practice group recalled significantly more facts than those in the non-retrieval practice group (*F*(1, 104) = 6.33, *p* = .01, *η*_p_^2^ = .06). There was no main effect of mind mapping (*F*(1, 104) = 1.93, *p* = .17), but an interaction was found between the conditions (*F*(1, 104) = 10.66, *p* = .001, *η*_p_^2^ = .09): Post-hoc testing indicated that using mind maps was more effective than not when in the non-retrieval condition (mean difference = 14.93%, *p* = .001), but offered no learning advantage when in the retrieval practice condition (mean difference = −6.16%, *p* = .19).

The covariate also had a significant influence on the outcome: Those who noted more facts tended to recall a higher percentage of facts later (*F*(1, 104) = 166.49, *p*<.001, *η*_p_^2^ = .61). Re-running the analysis without the covariate resulted in no main effect of either retrieval practice group (*F*(1, 105) = 2.16, *p* = .14) or mind map group (*F*(1, 105) = .01, *p* = .91), and no interaction (*F*(1, 105) = .68, *p* = .41). The retrieval practice effect was thus reliant on the inclusion as a covariate of the facts recorded in the learning phase, but the inclusion of the covariate was, in our view, justified: Taking into account baseline memory ability led to a more accurate estimation of the model results.

Whereas the data used here met the assumptions for ANCOVA, it could be argued that a logit analysis is more appropriate, since the test scores are binomial counts [26]. For this reason, we provide a secondary analysis using a generalized linear mixed model in the Text S1. This analysis produced the same pattern of results as the ANCOVA reported here (see Table S5).

### Discussion

Experiment 1 showed that the retrieval practice effect could reliably be found in primary school children, using similar methods to those of a previous study in adults [22]. Children in the retrieval practice group had significantly higher recall scores four days later than those in the non-retrieval group. The other study technique, mind mapping, did not exert a main effect on learning, but did improve learning compared to normal note-taking in the non-retrieval practice condition.

While they did not violate the assumption of normality (Kolmogorov-Smirnov test *D*(109) = .08, *p* = .09), we observed that the scores from the test in Experiment 1 were somewhat negatively skewed, indicating that the children did not, on average, find the tasks to be particularly challenging. In addition, the mean number of facts recorded in the learning phase was over 85%, indicating that the learning phase was longer than required for many to make a note of all of the facts. For these reasons, and to test whether the main results–the significant main effect of retrieval practice and its significant interaction with mind mapping–would replicate in a larger sample, we ran a second experiment in a different primary school.

In Experiment 2, we increased the difficulty of the tasks by increasing the number of facts on each factsheet, reducing the duration of the learning phase, and extending the interval between the learning and testing phases to one week for a first test, and five weeks for a second. The addition of the five-week test allowed us to assess longer-term outcomes, and examine forgetting across time.

## Experiment 2

### Method

#### Participants

Participants were 209 UK Primary School children (99 female), aged 8–12 years (*M* = 10.15 years, *SD* = 1.19), from Primaries 4, 5, 6, and 7 (two classes from each year; see Table S1 for numbers and ages per year) at Bruntsfield Primary School, Edinburgh. The experiment was approved by the Psychology Research Ethics Committee at the University of Edinburgh, and informed consent was obtained from the parent or guardian of each participating child.

#### Materials

Experiment 2 used very similar materials to Experiment 1 (see Fig. 1a and 1b), with more facts on the factsheets to increase the difficulty of the task. Sheets for Primary 4 children had eleven facts (∼one hundred words words), sheets for Primary 5 children had eighteen facts (∼one hundred and thirty-five words), and sheets for Primaries 6 and 7 had twenty-two facts (∼one hundred and fifty-five words). The text of each factsheet for each year group is provided in the Text S1.

#### Learning phase

The learning phase proceeded in the same manner as Experiment 1, with the same conditions (retrieval practice/non-retrieval, mind maps/no mind maps) but with a slight reduction in duration. It had the following structure: five minutes study/five minutes note-taking/three minutes study/three minutes note-taking. In this experiment, then, the learning phase lasted sixteen minutes, with the factsheets visible to the retrieval practice group for eight minutes only.

#### Testing phase

At one week and five weeks later, at the same time of day as the learning session had taken place, the children completed a written recall test, the same type as that in Experiment 1, in the same school classroom. The one-week test was administered by the experimenter, whereas the classroom teacher administered the five-week test; the pseudo-random order of the questions was different at each test (this order, along with a list of all questions for all year groups, is shown in the Text S1).

### Results

Again, a comparable number of children learned about each country (Senegal: fifty-two; South Korea: fifty-four; Peru: fifty; Iran: fifty-three; see Table S2 for the distributions of different sheets per experimental condition and per year group). Two note sheets from the learning phase were lost, leaving two hundred and seven sheets. An average of 75.95% of the facts were recorded on the note sheets; again there were no significant differences in this between conditions [2×2 ANOVA mean difference = 5.49% in favour of no mind mapping (*F*(1, 203) = 3.60, *p* = .06); mean difference = 1.25% in favour of non-retrieval (*F*(1, 203) = .17, *p* = .68)]. Table S3 details the performance in the learning phase by condition.

**Figure 2:**
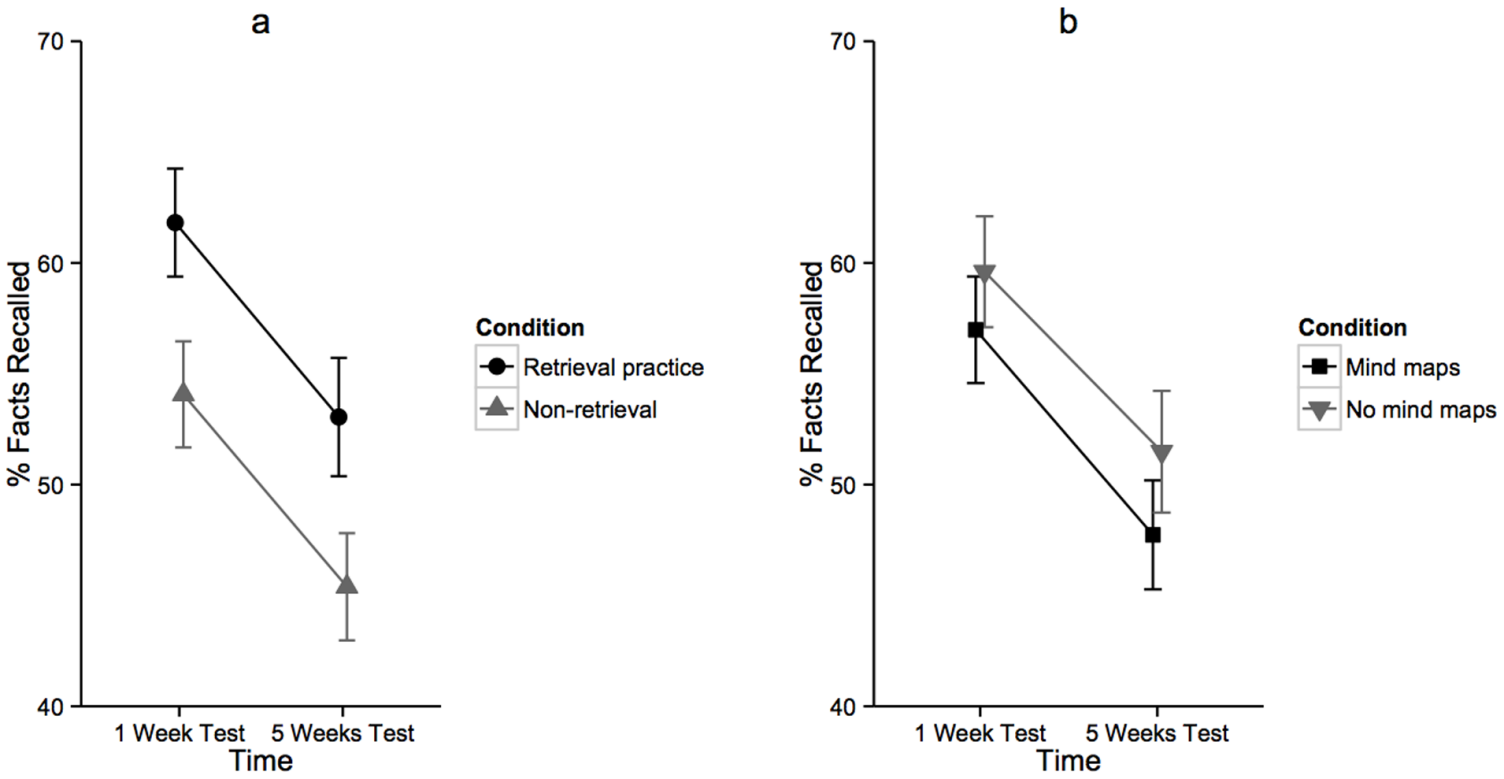
Percentage of facts recalled at the one- and five-week tests in Experiment 2 for (a) the retrieval practice and non-retrieval conditions and (b) the mind maps and no mind maps conditions. Error bars represent +/− 1 standard error.

Twenty-three children were unavailable at either the one- or the five-week tests, leaving one hundred and eighty-six children for the analysis of fact learning. Mean percentage recall scores for each condition, at each test, are shown in Table 2; Table S2 shows the number of participants from each year group in each condition. Figure 2 illustrates the mean recall results across the one- and five-week tests, first for the retrieval practice and non-retrieval conditions, and second for the mind maps and no mind maps conditions.

**Table 2:** Mean percentage scores (SDs) and sample sizes for the 1-week (white rows) and 5-week (shaded rows) recall tests (including only those who provided data at both tests).

	Weeks after learning	Mind maps	No mind maps	Total score	*N*
Retrieval	1	61.43 (24.48)	62.24 (24.21)	61.82 (24.23)	99
	5	52.82 (25.37)	53.30 (28.08)	54.07 (26.55)	
Non-retrieval	1	52.16 (22.83)	56.42 (21.77)	54.07 (22.33)	87
	5	42.22 (22.69)	49.29 (22.04)	45.39 (22.55)	
Total score	1	56.98 (23.04)	59.60 (23.19)	58.19 (23.62)	186
	5	47.73 (24.58)	51.48 (25.45)	49.47 (24.99)	
*N*		100	86	186	

To assess the effects of retrieval practice and mind mapping at both time-points, we ran a three-way (2×2×2) ANCOVA, including retrieval practice group (retrieval practice vs. non-retrieval) and mind mapping group (mind maps vs. no mind maps) as between-subjects factors and time of test (one or five weeks) as a within-subject factor. As in Experiment 1, we used multiple regression to identify related covariates: in this experiment, age, test type (Primary 4, Primary 5, or Primary 6/7) and facts recorded during the learning phase were all significantly related to the one-week score (all *p*-values<.001), and these variables plus sex were significantly related to the five-week score (for sex, *p*<.04; all other *p*-values<.001). Therefore, we included all four variables as covariates in the analysis.

Children in the retrieval practice group recalled significantly more facts than those in the non-retrieval practice group (*F*(1, 177) = 9.66, *p* = .002, *η*_p_^2^ = .05). The main effect of time was not significant (*F*(1, 177) = 1.83, *p* = .18), and there was no interaction of time with retrieval practice group (*F*(1, 177) = .001, *p* = .98) or with mind map group (*F*(1, 177) = .57, *p* = .45). There was no main effect of mind mapping (*F*(1, 177) = .18, *p* = .67) and, in contrast to Experiment 1, the interaction between retrieval practice and mind mapping was far from significance (*F*(1, 177) = .08, *p* = .78).

Regarding covariates, there was again a large and significant effect of facts recorded in the learning phase, with those initially recording more facts tending to recall a higher percentage later (*F*(1,177) = 97.48, *p*<.001, *η*_p_^2^ = .36) and there were also significant influences of age (F(1, 177) = 20.60, p<.001, *η*_p_^2^ = .10) and test type (F(1, 177) = 15.63, p<.001, *η*_p_^2^ = .08), such that older participants, and those with more facts to be remembered, tended to gain higher scores. There was no effect of sex (F(1, 177) = 2.52, p = .11).

To test whether there were any interactive effects of test type (that is, whether the number of facts was an influence on learning), in a further analysis we included this variable as a fixed effect, allowing it to interact with time and with the manipulated variables (retrieval practice and mind mapping group). No significant interactions were found between test type and time (*F*(1, 170) = 1.63, *p* = .20), retrieval practice group (F(1, 170) = .92, *p* = .40) or mind mapping group (*F*(1, 170) = .27, *p* = .77). All main effects and interactions between other variables remained significant or non-significant as in the original analysis. Thus, the effects of retrieval practice and mind mapping were comparable across all levels of the test.

In Experiment 1, the significant effect did not survive removal of the covariate. On the contrary, running the analysis with no covariates in Experiment 2 produced the same pattern of between-group results: significant effects of retrieval practice group (*F*(1, 182) = 4.73, *p* = .03, *η*_p_^2^ = .03) but not mind map group (*F*(1, 182) = .82, *p* = .37), and no significant interaction (*F*(1, 182) = .27, *p* = .60). Without covariates there was a main within-group effect of time (*F*(1, 182) = 65.88, *p*<.001, *η*_p_^2^ = .27), but no time×retrieval practice interaction (*F*(1, 182) = .02, *p* = .90), or time×mind map interaction (*F*(1, 182) = .26, *p* = .61).

As in Experiment 1, we report a secondary logit analysis of these results in the Text S1; results are shown in Table S6. Again, the results of this analysis did not appreciably differ from those of the ANCOVA reported here.

### Discussion

Experiment 2 was successful in increasing the difficulty of the tasks: Both the mean number of facts recorded on the sheets in the learning phase, and the mean number of facts recalled at the first test, were lower than in Experiment 1. It provided a replication of the main result from Experiment 1 in a larger sample, across a longer time interval, and robust to the inclusion or exclusion of covariates. Children in the retrieval practice condition recalled significantly more facts at the one- and five-week tests, albeit with a smaller effect size than for the four-day test administered in Experiment 1. Experiment 2 did not replicate the interaction between retrieval practice and mind mapping discovered in Experiment 1; mind mapping did not affect learning outcomes in either of the retrieval conditions.

## General Discussion

In two in-class experiments with primary school children, we compared the effects of retrieval practice and of mind mapping on later fact recall. The total time spent in the learning phase was the same in each condition, but children in the retrieval practice groups were exposed to the study materials for a far shorter time than those in the non-retrieval groups (in Experiment 2, exactly half as long). Despite this, children in the retrieval practice groups did not note down any fewer facts during learning, and subsequently recalled a significantly higher percentage of facts than those who did not use retrieval practice. This latter finding indicates that primary school teachers, like other educators, would benefit their pupils by using retrieval practice in the classroom.

Like Karpicke and Blunt [22], we found that retrieval practice improved fact recall, whereas a mapping technique had no main effects, even though the use of the latter is more widespread in schools. Our design additionally allowed for the evaluation of mapping *in combination* with retrieval practice, which we found did not have any special benefits for recall. However, in Experiment 1, we did find that retrieval practice and mind mapping interacted significantly, such that mind mapping was superior only in the non-retrieval condition. This finding was not replicated in our second experiment, where the interaction was very far from significance. Since the procedures were so similar across the two experiments, and since Experiment 2 had a larger sample size, the interaction in the initial experiment may have been a false-positive.

It should be noted that the mind mapping technique that our children used was necessarily simpler than the concept-mapping used by the undergraduate participants in the experiment by Karpicke and Blunt [22]. It may also be relevant that our children did not follow any specific recommendations for optimal mind mapping, such as the use of colour and pictures [16]. However, it is not clear to what extent such recommendations are evidence-based, and others advise that “…there is no necessity to retain an ideal structure or format” in mind mapping ([27], p. 282). Our over-riding concern was to test the mapping technique that was already being used regularly in the school we visited, with which the children were comfortable. Without exception, all children in the mind mapping groups in both experiments produced maps with the country name in the centre and radiating ‘spokes’ to either individual facts or groups of facts (a mind map of the latter type is shown in Figure 1). As noted above, very few studies have assessed the effects of mind mapping in young children; future experiments should manipulate aspects of the technique in line with popular recommendations [16] and test whether these provide mnemonic effects beyond the basic mind mapping employed in the present experiments, and whether they interact with other learning techniques such as retrieval practice. It may also be the case that children’s enjoyment of learning improves when techniques such as mind mapping are used, and this possibility should be studied, especially in the light of our findings that mind mapping had no detrimental effect on the number of facts initially noted (possibly due to the simplicity of the particular technique used), or on later recall.

The retrieval practice effects in both experiments were statistically significant, and above the recommended effect size threshold for practical significance [28]. The result from Experiment 1 is comparable to, though on the lower bound of, effect size estimates from previous studies in this age group: One previous paper [12], for example, found effect sizes of *d* = .64 and.54 (corresponding approximately to *η*_p_^2^ = .09 and.07, respectively) for retrieval practice on a test one day after learning, compared to our effect of *η*_p_^2^ = .06 for recall 4 days after learning. The overall effect in Experiment 2 was slightly smaller (*η*_p_^2^ = .05); the effect of retrieval practice thus if anything appeared to decline across the longer gap between the learning and testing phases. However, previous experiments (e.g. [9]) have found substantially larger effects with even longer learning-test intervals than in our study. This discrepancy may be explained by the younger age group involved in our study: Roediger & Karpicke [4] note that the retrieval practice effect may to some extent depend on age.

One potential limitation of Experiment 2 is that the same facts were tested at one and five weeks. The short-term test may thus have acted as retrieval practice for the longer-term test, boosting final performance. This may explain the finding that, unlike in some previous experiments (e.g. [29], though see [10]), retrieval practice did not slow forgetting across the four-week gap between tests in Experiment 2. However, any such influence should have raised the performance of the retrieval and non-retrieval groups equally. The difference between these groups was maintained at the longer-term test, indicating that using retrieval during initial learning is still beneficial in the longer-term, regardless of any intervening tests.

Two alternative explanations of the retrieval practice effects observed here should also be considered. First, since the participants in the retrieval group were able to look at their factsheet again after taking down some notes (on a mind map or otherwise), they potentially received feedback on their initial performance. This feedback could have alerted them to facts that they did not recall in the first note-taking period, or they could have repaired any errors they had made during the note-taking. Thus, the retrieval practice effect observed here might not have been a direct effect of retrieval, but a ‘mediated’ testing effect (see [4]), whereby the feedback, not the ‘testing effect’, aids later recall (this effect has also been described as “test-potentiated learning”, see e.g. [30]). However, participants in the retrieval group did not see their notes and the factsheet at the same time, and were not permitted to write anything during the second study period, which would impede direct comparisons between their notes and the factsheet. In addition, the non-retrieval group also had a period of restudy of the facts, where they could have reflected on the factsheets and received similar feedback on their note-taking performance; the retrieval group still outperformed the non-retrieval group. Neither of these points completely rule out a mediated testing effect, however; our design could not fully tease apart direct and indirect effects of retrieval practice.

Second, since our design precluded us from recording the number of facts recorded in the first note-taking period in the learning session, it may be the case that those in the non-retrieval groups–who had a somewhat easier task–recorded all of their facts during this period, and did not concentrate on the task during the second note-taking period. This would mean that the effective time on-task in the retrieval group was longer, explaining the better recall on the later test. However, if this interpretation were correct, we would expect children in the non-retrieval group to have written down more facts on average than the children using retrieval practice. As can be seen in Table S3, and in the ANOVA results reported above for both experiments, the total percentage of facts recalled in the learning phase was similar in both conditions, with the vast majority of children in both groups failing to record all of the facts. This implies that children in both groups were still working on their notes at the end of the study period, and that time on-task is not responsible for the retrieval effect.

### Conclusions

The two experiments reported here have practical implications for primary school teachers: using simple self-testing in the classroom by asking children to make notes on their learning materials from memory should significantly improve their recall of those materials several weeks later. The retrieval practice group in our Experiment 2 recalled over 8.5% more facts than the non-retrieval group, five weeks after the learning session. The popular technique of mind mapping, on the other hand, may be an interesting and enjoyable way for children to visually represent their learning, but teachers should not expect it to boost fact learning–at least of the type studied here–in the short- or long-term.

## Supporting Information

Table S1 Numbers and ages of children per year group in each of the two experiments.
(DOC)

Table S2 Distributions of each factsheet across the four cells of the experiment (upper part of table), and across year groups (lower part of table), for Experiments 1 and 2.
(DOC)

Table S3 Performance in the initial learning phase (percentage of all potential facts written on notes) for Experiment 1 (upper part of table) and Experiment 2 (lower part), broken down by condition.
(DOC)

Table S4 Numbers of participants in each year group in each cell of Experiments 1 and 2, only including participants who contributed data at all testing points.
(DOC)

Table S5 Results of the generalized linear mixed model for Experiment 1.
(DOC)

Table S6 Results of the generalized linear mixed model for Experiment 2.
(DOC)

Text S1 (DOC)
