# Supplementary material for: Retrieval Practice, with or without Mind Mapping, Boosts Fact Learning in Primary School Children
Source: PLoS One. 2013 Nov 12;8(11):e78976. doi: 10.1371/journal.pone.0078976 (PMC3827082; doi:10.1371/journal.pone.0078976)
Supplement: Table S1 — Numbers and ages of children per year group in each of the two experiments. (DOC) [file pone.0078976.s001.doc]

|  | Experiment 1 | |  | Experiment 2 | | | |
| --- | --- | --- | --- | --- | --- | --- | --- |
|  | Primary 5 | Primary 7 |  | Primary 4 | Primary 5 | Primary 6 | Primary 7 |
| *n* | 59 | 50 |  | 65 | 47 | 50 | 47 |
| Mean age in years (*SD*) | 9.35 (.31) | 11.39 (.32) |  | 8.82 (.28) | 9.71 (.29) | 10.68 (.32) | 11.86 (.32) |
