# Supplementary material for: Retrieval Practice, with or without Mind Mapping, Boosts Fact Learning in Primary School Children
Source: PLoS One. 2013 Nov 12;8(11):e78976. doi: 10.1371/journal.pone.0078976 (PMC3827082; doi:10.1371/journal.pone.0078976)
Supplement: Table S2 — Distributions of each factsheet across the four cells of the experiment (upper part of table), and across year groups (lower part of table), for Experiments 1 and 2. (DOC) [file pone.0078976.s002.doc]

| Country on Factsheet | Frequency per condition in Experiment 1 | | | |  | Frequency per condition in Experiment 2 | | | |
| --- | --- | --- | --- | --- | --- | --- | --- | --- | --- |
|  | Retrieval Practice | | Non-retrieval | |  | Retrieval Practice | | Non-retrieval | |
|  | Mind Maps | No Mind Maps | Mind Maps | No Mind Maps |  | Mind Maps | No Mind Maps | Mind Maps | No Mind Maps |
| Senegal | 8 | 8 | 7 | 8 |  | 14 | 11 | 15 | 12 |
| South Korea | 5 | 7 | 10 | 6 |  | 14 | 13 | 14 | 13 |
| Iran | 6 | 7 | 7 | 5 |  | 16 | 14 | 12 | 11 |
| Peru | 7 | 4 | 7 | 7 |  | 13 | 12 | 14 | 11 |
| Country on Factsheet | Frequency per year group in Experiment 1 | | | |  | Frequency per year group in Experiment 2 | | | |
|  | Primary 5 | | Primary 7 | |  | Primary 4 | Primary 5 | Primary 6 | Primary 7 |
| Senegal | 17 | | 15 | |  | 17 | 13 | 10 | 12 |
| South Korea | 15 | | 13 | |  | 16 | 12 | 14 | 12 |
| Iran | 13 | | 11 | |  | 16 | 11 | 14 | 12 |
| Peru | 14 | | 11 | |  | 16 | 11 | 12 | 11 |
