# Supplementary material for: Retrieval Practice, with or without Mind Mapping, Boosts Fact Learning in Primary School Children
Source: PLoS One. 2013 Nov 12;8(11):e78976. doi: 10.1371/journal.pone.0078976 (PMC3827082; doi:10.1371/journal.pone.0078976)
Supplement: Table S3 — Performance in the initial learning phase (percentage of all potential facts written on notes) for Experiment 1 (upper part of table) and Experiment 2 (lower part), broken down by condition. (DOC) [file pone.0078976.s003.doc]

| Experiment 1 |  | Mind maps | No mind maps | Total score |
| --- | --- | --- | --- | --- |
|  | Retrieval | 82.87 (17.55) | 81.95 (19.12) | 82.40 (18.18) |
|  | Non-retrieval | 77.47 (24.63) | 88.29 (19.00) | 83.26 (22.27) |
|  | Total score | 80.17 (21.35) | 85.34 (19.15) | 82.85 (20.31) |
| Experiment 2 |  | Mind maps | No mind maps | Total score |
|  | Retrieval | 75.50 (18.85) | 75.67 (22.25) | 75.58 (20.42) |
|  | Non-retrieval | 71.42 (25.70) | 82.24 (18.28) | 76.35 (23.16) |
|  | Total score | 73.48 (22.50) | 78.81 (20.60) | 75.95 (21.75) |
