# Supplementary material for: Retrieval Practice, with or without Mind Mapping, Boosts Fact Learning in Primary School Children
Source: PLoS One. 2013 Nov 12;8(11):e78976. doi: 10.1371/journal.pone.0078976 (PMC3827082; doi:10.1371/journal.pone.0078976)
Supplement: Table S4 — Numbers of participants in each year group in each cell of Experiments 1 and 2, only including participants who contributed data at all testing points. (DOC) [file pone.0078976.s004.doc]

| Experiment |  | Year group | Mind maps | No mind maps | Total |
| --- | --- | --- | --- | --- | --- |
| 1 | Retrieval | Primary 5 | 15 | 13 | 28 |
|  |  | Primary 7 | 11 | 13 | 24 |
|  | Non-retrieval | Primary 5 | 13 | 17 | 30 |
|  |  | Primary 7 | 13 | 13 | 26 |
|  | Total | Primary 5 | 28 | 30 | 38 |
|  |  | Primary 7 | 24 | 26 | 50 |
| 2 | Retrieval | Primary 4 | 17 | 15 | 32 |
|  |  | Primary 5 | 9 | 13 | 22 |
|  |  | Primary 6 | 11 | 14 | 25 |
|  |  | Primary 7 | 12 | 8 | 20 |
|  | Non-retrieval | Primary 4 | 14 | 14 | 28 |
|  |  | Primary 5 | 11 | 10 | 21 |
|  |  | Primary 6 | 9 | 11 | 20 |
|  |  | Primary 7 | 12 | 6 | 18 |
|  | Total | Primary 4 | 31 | 29 | 60 |
|  |  | Primary 5 | 20 | 23 | 43 |
|  |  | Primary 6 | 20 | 25 | 45 |
|  |  | Primary 7 | 24 | 14 | 38 |
