# Supplementary material for: Retrieval Practice, with or without Mind Mapping, Boosts Fact Learning in Primary School Children
Source: PLoS One. 2013 Nov 12;8(11):e78976. doi: 10.1371/journal.pone.0078976 (PMC3827082; doi:10.1371/journal.pone.0078976)
Supplement: Table S5 — Results of the generalized linear mixed model for Experiment 1. (DOC) [file pone.0078976.s005.doc]

| Fixed effects | Coefficient | SE | *z* | *p* |
| --- | --- | --- | --- | --- |
| (Intercept) | 1.82 | .23 | 8.00 | < .001 |
| Group – Non-retrieval | −1.45 | .30 | −4.75 | < .001 |
| Group – Mind Maps | −.58 | .31 | −1.85 | .06 |
| Facts recorded in learning session | 1.26 | .11 | 11.12 | < .001 |
| Retrieval × mind map interaction | 1.61 | .44 | 3.69 | < .001 |
|  |  |  |  |  |
| Random effects | Variance | SD | No. observations |  |
| ID (Intercept) | .90 | .95 | 109 |  |
