# Supplementary material for: Retrieval Practice, with or without Mind Mapping, Boosts Fact Learning in Primary School Children
Source: PLoS One. 2013 Nov 12;8(11):e78976. doi: 10.1371/journal.pone.0078976 (PMC3827082; doi:10.1371/journal.pone.0078976)
Supplement: Table S6 — Results of the generalized linear mixed model for Experiment 2. (DOC) [file pone.0078976.s006.doc]

| Fixed effects | Coefficient | SE | *z* | *p* |
| --- | --- | --- | --- | --- |
| (Intercept) | −4.09 | 1.13 | −3.61 | < .001 |
| Time – 5 weeks | −.51 | .10 | −4.98 | < .001 |
| Group – Non-retrieval | −.45 | .20 | −2.32 | .02 |
| Group – Mind Maps | −.18 | .19 | −.98 | .33 |
| Age | .58 | .13 | 4.53 | < .001 |
| Quiz – Primary 5 | −.81 | .21 | −3.85 | < .001 |
| Quiz – Primary 6/7 | −1.86 | .34 | −5.48 | < .001 |
| Facts recorded in learning session | .67 | .07 | 10.27 | < .001 |
| Time × retrieval interaction | .08 | .15 | .51 | .60 |
| Time × mind map interaction | .11 | .14 | .81 | .42 |
| Retrieval × mind map interaction | .14 | .26 | .53 | .76 |
| Time × retrieval × mind map interaction | −.17 | .20 | −.82 | .41 |
|  |  |  |  |  |
| Random effects | Variance | SD | No. observations |  |
| ID (Intercept) | .69 | .83 | 201 |  |
| Time - 5 Weeks | .17 | .41 | 386 |  |
