# Supplementary material for: Retrieval Practice, with or without Mind Mapping, Boosts Fact Learning in Primary School Children
Source: PLoS One. 2013 Nov 12;8(11):e78976. doi: 10.1371/journal.pone.0078976 (PMC3827082; doi:10.1371/journal.pone.0078976)
Supplement: Text S1 — (DOC) [file pone.0078976.s007.doc]

**Supporting Information**

**Retrieval practice, with or without mind mapping, boosts fact learning in primary school children**

Stuart J. Ritchie, Sergio Della Sala, & Robert D. McIntosh

*The University of Edinburgh*

This supporting information document gives the full text of the factsheets (from the learning phases) and the test questions (from the testing phases) used in both experiments. It includes extra information in table form on the numbers and ages of children in each age group for both experiments (Table S1), along with the distributions of each factsheet across each condition (Table S2), details of performance on the initial task in the learning phase (Table S3), and the number of participants in each cell of the experiment from each year (Table S4). Finally, it provides an alternative analysis of the main results in both experiments, using mixed-effects modeling.

**Full factsheet texts**

Below is the text from all the factsheets read by the children in the learning phases for Experiments 1 and 2, for each age group.

*Text of all fact sheets for Primary 5 in Experiment 1.*

**1) Senegal**

**The country.** Senegal is a country in Africa. In a list of the world’s biggest countries, Senegal is number 67. The capital city of Senegal is called Dakar. It takes 6 hours to fly there from Edinburgh.

**The people.** Most people in Senegal live in the countryside, and most of them follow the religion of Islam.

**What is it like to live there?** The weather in Senegal is hot all year round. Senegal is famous for its music.

**What do they make there?** Two things made in Senegal are peanuts and coffee, which are sold to people in other countries.

**2) South Korea**

**The country.** South Korea is a country in Asia. In a list of the world’s biggest countries, South Korea is number 109. The capital city of South Korea is called Seoul. It takes 10 hours to fly there from Edinburgh.

**The people.** Most people in South Korea live in cities, and most of them do not have a religion.

**What is it like to live there?** The weather in South Korea is hot and wet. South Korea is famous for its computers.

**What do they make there?** Two things made in South Korea are boats and robots, which are sold to people in other countries.

**3) Peru**

**The country.** Peru is a country in South America. In a list of the world’s biggest countries, Peru is number 20. The capital city of Peru is called Lima. It takes 12 hours to fly there from Edinburgh.

**The people.** Most people in Peru live in cities, and most of them follow the religion of Christianity.

**What is it like to live there?** The weather in Peru is hot all year round. Peru is famous for its mountains.

**What do they make?** Two things made in Peru are fish and gold, which are sold to people in other countries.

**4) Iran**

**The country.** Iran is a country in Asia. In a list of the world’s biggest countries, Iran is number 18. The capital city of Iran is called Tehran. It takes 6 hours to fly there from Edinburgh.

**The people.** Most people in Iran live in cities, and most of them follow the religion of Islam.

**What is it like to live there?** The weather in Iran is hot and dry. Iran is famous for its history.

**What do they make?** Two things made in Iran are oil and carpets, which are sold to people in other countries.

*Text of all fact sheets for Primary 7 in Experiment 1.*

**1) Senegal**

**The country.** Senegal is a country in Africa. Senegal is a medium-sized country, and in a list of the world’s biggest countries, Senegal comes 67^th^. The capital city of Senegal is called Dakar. It takes 6 hours to fly there from Edinburgh.
**The people.** Thirteen million people live in Senegal, and most of them live in the countryside. Most of the people in Senegal follow the religion of Islam.
**What is it like to live there?** Senegal has a tropical climate, meaning it is hot all year round. Senegal is famous for its music.
**What do they make there?** One kind of Senegalese food is yassa, which is made of fish and garlic. Two things produced in Senegal are peanuts and coffee, which are sold to people in other countries.

**2) South Korea**

**The country.** South Korea is a country in Asia. South Korea is a small country, and in a list of the world’s biggest countries, South Korea comes 109^th^. The capital city of South Korea is called Seoul. It takes 10 hours to fly there from Edinburgh.
**The people.** Forty-five million people live in South Korea, and most of them live in cities. Most of the people in South Korea do not follow any religion.
**What is it like to live there?** South Korea has a humid climate, meaning it is hot and wet. South Korea is famous for its technology.
**What do they make there?** One kind of South Korean food is kimchi, which is made of vegetables and spices. Two things produced in South Korea are ships and robots, which are sold to people in other countries.

**3) Peru**

**The country.** Peru is a country in South America. Peru is a large country, and in a list of the world’s biggest countries, Peru comes 20^th^. The capital city of Peru is called Lima. It takes 12 hours to fly there from Edinburgh.
**The people.** Thirty million people live in Peru, and most of them live in cities. Most of the people in Peru follow the religion of Christianity.
**What is it like to live there?** Peru has a tropical climate, meaning it is hot all year round. Peru is famous for its mountains.
**What do they make?** One kind of Peruvian food is pachamanca, which is made from meat and spices. Two things produced in Peru are fish and gold, which are sold to people in other countries.

**4)Iran**

**The country.** Iran is a country in Asia. Iran is a large country, and in a list of the world’s biggest countries, Iran comes 18^th^. The capital city of Iran is called Tehran. It takes 6 hours to fly there from Edinburgh airport.
**The people.** Seventy-five million people live in Iran, and most of them live in cities. Most of the people in Iran follow the religion of Islam.
**What is it like to live there?** Iran has an arid climate, which means it is hot and dry. Iran is famous for its history.
**What do they make?** One kind of Iranian food is tah-chin, which is made from rice and chicken. Two things produced in Iran are oil and carpets, which are sold to people in other countries.

*Text of all fact sheets for Primary 5 in Experiment 2 (nb. fact sheets for Primary 4 were identical to those used for Primary 5 in Experiment 1).*

**1) Senegal**

**The country.** Senegal is a country in Africa. In a list of the world’s biggest countries, Senegal comes 67^th^. The capital city of Senegal is Dakar. It takes 6 hours to fly there from Edinburgh.
**The people.** Thirteen million people live in Senegal, and most of them live in the countryside. Most of the people in Senegal follow the religion of Islam.
**What is it like to live there?** Senegal has a tropical climate, meaning it is hot all year round. Senegal is famous for its music and its football. The money used in Senegal is called the Franc.
**What do they make there?** One kind of Senegalese food is yassa, which is made of fish and garlic. Two things made in Senegal are peanuts and coffee, which are sold to people in other countries.

**2) South Korea**

**The country.** South Korea is a country in Asia. In a list of the world’s biggest countries, South Korea comes 109^th^. The capital city of South Korea is called Seoul. It takes 10 hours to fly there from Edinburgh.
**The people.** Forty-five million people live in South Korea, and most of them live in cities. Most of the people in South Korea do not follow any religion.
**What is it like to live there?** South Korea has a humid climate, meaning it is hot and wet. South Korea is famous for its technology and its martial arts. The money used in South Korea is called the Won.
**What do they make there?** One kind of South Korean food is kimchi, which is made of vegetables and spices. Two things made in South Korea are ships and robots, which are sold to people in other countries.

**3) Peru**

**The country.** Peru is a country in South America. In a list of the world’s biggest countries, Peru comes 20^th^. The capital city of Peru is called Lima. It takes 12 hours to fly there from Edinburgh.
**The people.** Thirty million people live in Peru, and most of them live in cities. Most of the people in Peru follow the religion of Christianity.
**What is it like to live there?** Peru has a tropical climate, meaning it is hot all year round. Peru is famous for its mountains and its animals. The money used in Peru is called the Sol.
**What do they make?** One kind of Peruvian food is pachamanca, which is made from meat and spices. Two things made in Peru are fish and gold, which are sold to people in other countries.

**4) Iran**

**The country.** Iran is a country in the Middle East. In a list of the world’s biggest countries, Iran comes 18^th^. The capital city of Iran is called Tehran. It takes 6 hours to fly there from Edinburgh airport.
**The people.** Seventy-five million people live in Iran, and most of them live in cities. Most of the people in Iran follow the religion of Islam.
**What is it like to live there?** Iran has an arid climate, which means it is hot and dry. Iran is famous for its history and its culture. The money used in Iran is called the Rial.
**What do they make?** One kind of Iranian food is tah-chin, which is made from rice and chicken. Two things made in Iran are oil and carpets, which are sold to people in other countries.

*Text of all fact sheets for Primary 6 and 7 in Experiment 2.*

**1) Senegal**

**The country.** Senegal is a country in Africa. In a list of the world’s biggest countries, Senegal comes 67^th^. The capital city of Senegal is Dakar. It takes 6 hours to fly there from Edinburgh.
**The people.** Thirteen million people live in Senegal, and most of them live in the countryside. Most of the people in Senegal follow the religion of Islam, and the language most of them speak is French. The leader of Senegal is called Macky Sall.
**What is it like to live there?** Senegal has a tropical climate, meaning it is hot all year round. Senegal is famous for its music and its football. The money used in Senegal is called the Franc.
**What do they make there?** One kind of Senegalese food is yassa, which is made of fish and garlic. Three things produced in Senegal are cotton, peanuts, and coffee, which are sold to people in other countries.
 **2) South Korea
The country.** South Korea is a country in Asia. In a list of the world’s biggest countries, South Korea comes 109^th^. The capital city of South Korea is called Seoul. It takes 10 hours to fly there from Edinburgh.
**The people.** Forty-five million people live in South Korea, and most of them live in cities. Most of the people in South Korea do not follow any religion, and the language most of them speak is Korean. The leader of South Korea is called Lee Myung-Bak.
**What is it like to live there?** South Korea has a humid climate, meaning it is hot and wet. South Korea is famous for its technology and its martial arts. The money used in South Korea is called the Won.
**What do they make there?** One kind of South Korean food is kimchi, which is made of vegetables and spices. Three things produced in South Korea are ships, cars, and robots, which are sold to people in other countries.

**3) Peru
The country.** Peru is a country in South America. In a list of the world’s biggest countries, Peru comes 20^th^. The capital city of Peru is called Lima. It takes 12 hours to fly there from Edinburgh.
**The people.** Thirty million people live in Peru, and most of them live in cities. Most of the people in Peru follow the religion of Christianity, and the language most of them speak is Spanish. The leader of Peru is called Ollanta Humala.
**What is it like to live there?** Peru has a tropical climate, meaning it is hot all year round. Peru is famous for its mountains and its animals. The money used in Peru is called the Sol.
**What do they make?** One kind of Peruvian food is pachamanca, which is made from meat and spices. Three things produced in Peru are wood, fish, and gold, which are sold to people in other countries.

**4)Iran
The country.** Iran is a country in the Middle East. In a list of the world’s biggest countries, Iran comes 18^th^. The capital city of Iran is called Tehran. It takes 6 hours to fly there from Edinburgh airport.
**The people.** Seventy-five million people live in Iran, and most of them live in cities. Most of the people in Iran follow the religion of Islam and the language most of them speak is Persian. The leader of Iran is called Ali Khamenei.
**What is it like to live there?** Iran has an arid climate, which means it is hot and dry. Iran is famous for its history and its culture. The money used in Iran is called the Rial.
**What do they make?** One kind of Iranian food is tah-chin, which is made from rice and chicken. Three things produced in Iran are oil, nuts, and carpets, which are sold to people in other countries.

**Testing phase questions**

Below is a list of all the questions read to the children in the testing phases for Experiments 1 and 2. The questions apply to each of the four factsheets, so they could be administered to all children *en masse*.

*Test questions used in Experiment 1 for Primary 5.*

1) What is the name of the country you learned about?
2) Name two things made in the country that are sold to other countries.
3) Where is the country?
4) Which number is the country in a list of world’s biggest countries?
5) What is the name of the country’s capital city?
6) How many hours does it take to fly there from Edinburgh?
7) What is the weather like in the country?
8) What is the country famous for?
9) Where do most people in the country live?
10) Do most people in the country have a religion? If they do, which one is it?

*Test questions used in Experiment 1 for Primary 7.*

1) What is the name of the country you learned about?
2) Name a kind of food from the country.
3) What is the food made of?
4) Name two things made in the country that are sold to people in other countries.
5) Where is the country?
6) Which number is the country in a list of the world’s biggest countries?
7) What is the name of the country’s capital city?
8) How many hours does it take to fly there from Edinburgh?
9) What is the climate of the country, and what does this mean?
10) What is the country famous for?
11) How many people live in the country, in millions?
12) Where do most of the people in the country live?
13) Do most people in the country have a religion? If they do, which one is it?

*Test questions used at one week in Experiment 2 for Primary 5 (n.b. questions for Primary 4 were identical to those for Primary 5 in Experiment 1), followed by the order of the questions at the five-week test.*

1) What is the name of the country you learned about last week?

2) Where is the country?

3) Where does the country come in a list of the world’s biggest countries?

4) How many people live in the country?

5) Where do most of them live?

6) Name two things the country is famous for.

7) What is the name of the money used in the country?

8) Which religion do most of the people follow in the country?

9) What kind of climate does the country have, and what does this mean?

10) Name two things that are made in the country.

11) What is the name of the country’s capital city?

12) How long would it take to fly there from Edinburgh?

13) Name a kind of food made in the country.

14) What two things is the food made of?

Order of the above questions at the five-week test: 1, 2, 5, 8, 3, 11, 10, 13, 14, 5, 6, 7, 4, 9.

*Test questions used (for all countries simultaneously) at one week in Experiment 2 for Primary 6 and 7, followed by the order of the questions at the five-week test.*

1) What is the name of the country you learned about last week?

2) Where is the country?

3) Where does the country come in a list of the world’s biggest countries?

4) How many people live in the country?

5) Where do most of them live?

6) Name two things the country is famous for.

7) Which language do most people speak in the country?

8) What is the name of the money used in the country?

9) Which religion do most of the people follow in the country?

10) What is the name of the country’s leader?

11) What kind of climate does the country have, and what does this mean?

12) Name three things that are produced in the country.

13) What is the name of the country’s capital city?

14) How long would it take to fly there from Edinburgh?

15) Name a kind of food made in the country.

16) What two things is the food made of?

Order of the above questions at the five-week test: 1, 2, 4, 3, 11, 10, 5, 6, 9, 15, 16, 12, 13, 8, 14, 7.

**Supplementary analyses**

As noted in the main article, we view the ANCOVA we used as the most transparent and straightforward way to analyze our results (especially given that the data from both experiments fit the assumptions of that method). Nevertheless, it could be argued that this analysis is not optimal since the data from the tests is in the form of binomial counts: that is, it records how many test questions, which are either correct or incorrect, each participant answered correctly [26]. In addition, since each year group were administered tests with different numbers of answers (more difficult tests for older children), we had more information for the older children, and thus more reliable results. Collapsing the scores across these tests into *z*-scores, as we did for the ANCOVA above, results in a loss of this extra information.

Here, we provide a supplementary analysis of the data from both of our experiments, and instead of using ANCOVA we use a generalized linear mixed-effects model that takes into account the binomial nature of the data and the different tests. For both experiments, the analysis was carried out using the ‘lmer’ command in the R package ‘lme4’ [S1].

**Experiment 1**

For the first experiment, we ran a generalized linear mixed-effects model for binomial counts. Instead of using the *z*-score of test ability, the dependent variables were the number of facts correctly written down, and the number of facts incorrectly written down or not written down. The model allowed for main effects of, and the interaction between, retrieval practice group and mind mapping group, and controlled for the number of facts recorded in the learning session. A random effect of participant ID number was added to indicate that participants will differ in their ability to recall facts.

The results of this analysis are shown in Table S5. The pattern of results was similar to that in the ANCOVA analysis: a significant effect of retrieval practice group, no significant effect of mind mapping group, and a significant retrieval group × mind mapping group interaction, along with a significant influence of the single covariate, number of facts recorded during the learning session.

**Experiment 2**

For experiment 2, the analysis was altered to take into account the multiple measurement occasions. A similar model to Experiment 1 was run, this time also including the within-subjects factor of time (1 or 5 weeks after the learning phase) and the covariates of age, and test type (Primary 4, Primary 5, or Primary 6/7). The random effect in this model involved each participant's ID number and time, and thus allowed for differences in individual ability to recall facts across time.

Table S6 shows the results of this model, again indicating that the results in this supplementary analysis did not differ strongly from those in the main ANCOVA analysis. Significant main effects were found for retrieval practice group, but not for mind mapping group, and there was no significant interaction found between the groups. Time had a significant effect, but did not interact with either of the conditions.

**Reference**

S1. Bates D (2005) Fitting linear mixed models in R: Using the lme4 package. *R News*, 5: 27–30.
